# EFL Students’ Academic Buoyancy: Does Academic Motivation and Interest Matter?

**DOI:** 10.3389/fpsyg.2022.858054

**Published:** 2022-03-17

**Authors:** Xin Xu, Bin Wang

**Affiliations:** ^1^School of Foreign Languages, Shanghai Jiao Tong University, Shanghai, China; ^2^College of Chinese Language and Literature, Qufu Normal University, Qufu, China

**Keywords:** academic buoyancy, academic motivation, foreign language learning, interest, academic studies

## Abstract

The way students are treated by their teachers have been proven to play a paramount role in students’ performance. Either teachers’ academic buoyancy or academic motivation may change students’ mindset about learning a language, leading to facilitating this process or making it even more demanding than it seems. Considering that, it can be taken into account how important teachers’ academic buoyancy would be and its mediators such as academic motivation should draw precise attention to themselves. However, a few studies have been conducted about this pivotal role so far. To fill this gap, this review paper provides a glimpse at the underlying roles of academic buoyancy and academic motivation and interest. Furthermore, it presents the definitions and applications of each construct. Finally, some implications and future recommendations are put forward to enthusiastic scholars.

## Introduction

Teaching has long been perceived as a stressful, yet rewarding job, in which teachers’ and students’ emotions and inner thoughts have an enormous impact on the academic aspect of their lives (Zhang, 2021). Because motivation and interests play a pivotal role in students’ academic buoyancy, studies covering such topics are of paramount significance. Previous studies have mostly highlighted motivation (Dewaele, 2015; MacIntyre et al., 2019; Wang et al., 2021), but attention should be drawn to the fact that few studies have been conducted to emphasize the significance of learners’ buoyancy and its relevance to students’ motivation and interests in instructional domains. Furthermore, what makes it an important subject to be examined is its predominant role in learners’ motivation and how Buoyant a student can be when they are highly motivated. This study hence aims to highlight the association between EFL learners’ academic buoyancy and their motivation and interests. In an effort to do that, first of all, two variables have been defined and their importance has been highlighted. Academic buoyancy then has been dealt with as well as its relationship with EFL learners’ motivation and interests, and some implications and further recommendations for future studies have been finally put forward.

## Background

### Positive Psychology

With the birth of positive psychology in the domain of language education (MacIntyre and Gregersen, 2021), researchers’ attention has been focused on the fact that it is not just negative emotions such as boredom, burnout, and anxiety that may affect the process of learning and teaching; however, both negative and positive emotions can be taken into account due highly to the fact that they cannot be separated in real life. As pinpointed by Gregersen (2013), positive psychology not only facilitates the learning and teaching process but also makes it more meaningful and enjoyable. It helps students to be more resilient and to be faced with the challenges of L2 learning. Furthermore, friendly education, being academically engaged, being passionate, persistent, and diligent while one is achieving his goals which are known as grit, being resilient, regulating emotions, wellbeing, and enjoyment are the tenets of positive psychology through which the process of learning and teaching can be flourished.

### Academic Buoyancy

Buoyancy originated from Positive Psychology which has placed its accentuation on the role of emotions in instruction. What students experience in terms of academic aspects provide them with the opportunities to become more flexible. However, students are also pressurized enough by the exams for which they have to study, by homework assignments that need to be done, by poor grades they might be given, and by the deadlines that are expected to be met (Martin and Marsh, 2008). There is, therefore, a pressing issue that should be taken into consideration to which buoyancy can be viewed as a solution. The more buoyant students are, the more motivated they become to follow what they do with ardor. According to Yun et al. (2018), buoyancy can be conceptualized as a way through which the apogees and perigees of learning a language can be negotiated and everyday challenges, facing while learning a new language, can be addressed. As it has been claimed by Martin and Marsh (2008), academic buoyancy is different from what has been known as resilience, as a traditional construct. As discussed in the following studies, resilience is concerned with terrible situations that one may encounter during their lives, such as disabilities, while buoyancy focuses on daily controversies, pressures, and setbacks. Furthermore, as opposed to resilience, buoyancy has an inclination to bear a proactive approach toward these happenings than a reactive one. In other words, those who are buoyant do not just react to an adverse situation but try to enhance their wellbeing with the passage of time and as a result of which psychological growth can be achieved. Likewise, immunity is said to be similar to buoyancy through which defensive mechanisms can be used for the challenges, perturbation, and damages to be minimized (Hiver, 2017). Hardiness is another synonym for buoyancy which is a personality trait that enables people to reduce the effects of stress while performing something (Hiver and Dörnyei, 2017). Similarly, another synonymous term for buoyancy is coping which refers to the techniques that are exploited which can either alleviate the sources of stress or modify the way they are understood by the individuals (Somerfield and McCrae, 2000).

Following the literacy on EFL learners’ buoyancy, it was also pinpointed that buoyant students are cable to monitor their experiences which can be contrasted with the rest in society, leading to being autonomous, determined, and self-assurance (Martin, 2013; Martin et al., 2013). There is robust evidence to support the view that both educational and psychological outcomes are influenced by academic buoyancy. The extent to which one can enjoy the class falls under the category of the former one, educational outcomes, and self-efficacy and self-esteem are the exemplifications of the latter one.

Another study carried out by Yun et al. (2018) evaluated learners’ academic buoyancy, L2 achievements, and GPA (grade point average) by distributing the questionnaires to 787 language learners, studying in college in South Korea. The results revealed that buoyancy fundamentally anticipated both L2 accomplishment and GPA. In a study conducted by Jahedizadeh et al. (2019), the relationship between EFL learners’ buoyancy and three variables including, gender, GPA, and their educational levels was investigated through a newly devised questionnaire. The above-mentioned questionnaire was composed of 27 items, measuring four aspects of buoyancy which are as follows: the capacity to endure in a relatively ongoing way across various domains of life, sustainability, regularity conformity, positive personal competency, and positively accepting academic life (Martin et al., 2013; Putwain et al., 2015) similarly declared that academic buoyancy is negatively affected by worry. To put it simply, high motivation can be found with buoyant students who profoundly believe in themselves. It was also demonstrated that learners’ academic achievements are predicted by academic buoyancy. What is vividly apparent from studies under the subject of buoyancy is that there is a strong connection between academic learning and buoyancy (Yun et al., 2018). This study, hence, aims to find the relationship between academic buoyancy in EFL students and their level of motivation and interests since to the best knowledge of the researcher; few studies have been conducted so far to focus on EFL students’ academic buoyancy. It was mostly done in other contexts, to name a few, high schools for other subjects.

### EFL Learners’ Academic Motivation and Interest

Demotivation is an ongoing and pervasive problem facing students which contribute to not being enthusiastic to pursue their education. In addition, their academic buoyancy has remained relatively unchanged throughout their studies. Students with less motivation and interests are, therefore, markedly less happy than those who are highly motivated.

Motivation is thought of as an indicative factor that helps students to go up the ladder of success and improve their learning throughout the class (Meng, 2021). A study conducted by Liu and Chiang (2019) has shown the impact of both family and teachers on how learners’ motivation is shaped throughout the process of learning. It was done in a Chinese context, considering three subjects, math, Chinese, and English. It indicated that the above-determining factors predict motivation in students; however, EFL learners’ motivation to learn English is highly affected by their family background. It was also mentioned that educational inequality arises as a result of the role played by teachers and families. Another study showed the relationship between positive psychology and individuals’ development in both learning and teaching contexts (Wang et al., 2021). In another study carried out by Xie and Derakhshan (2021, p. 1), the role of teacher interpersonal variables namely, “teacher care, credibility, clarity, rapport, immediacy and stroke in students’ academic engagement, success, and motivation” has been discussed and it was revealed that those variables are the facilitators of students’ academic outcomes. In a study done by Ulfa and Bania (2019), both the impact of intrinsic and extrinsic motivation in language learning has been evaluated in EFL learners. The results revealed that extrinsic motivation is extremely influenced by the teachers and the way the learners are treated by them, while learners’ intrinsic motivation is highly impacted by the learning goals set by learners themselves. Learners’ interests fall under the category of the cognitive engagement domain which includes motivation, diligence, and strategy use. It, additionally, is a positive orientation toward an activity that brings value to what he does (Reschly and Christenson, 2006; Rotgans and Schmidt, 2011). It can be taken as an example when a person is keen to read about life in the middle ages, he tries to watch some movies about this topic, read some books, engage, and reengage in the activity; the driving force behind doing all these activities can be called “interest” (Ainley, 2012). As it has been told by Frydenberg et al. (2005), energy and direction are defined with the help of motivation. In other words, motivation is the reason behind how we behave, what we do, and why we do it. Being motivated does not necessarily mean that one can be actively engaged; consequently, it is not sufficient for engagement (Appleton et al., 2006; Wang and Guan, 2020).

From an educational point of view, motivation can be classified into three categories which goes as follows: when students believe in their abilities while doing a task (abilities), the reasons behind carrying out a task, and the goals set by the individuals to be achieved (strategies), how to respond emotionally as one is doing the task (behavior). Motivation has been also categorized into two other major divisions: external and internal. While the former one is concerned with setting an autonomous goal that requires a specific activity such as factors related to academic contexts (courses they take, the exams they take, rewards, and feedback), social factors (teachers, family members, and classmates), college environment (the physical environment and volunteering), extracurricular activities (sports activities), and the latter one refers to enough incentives to carry out a task, such as the characteristics of the students (social class) and the beliefs that students hold. Academic achievement has been said to be correlated with academic motivation because those students who are capable of completing a task are more liable to take some strategies into account and behave in a way that helps them achieve their goals. Academic motivation has a positive impact on the learning process. It causes students to put effort into practice so as to do the difficult task however hard it seems (Schunk, 1991; Van Etten et al., 2008).

### The Relationship Between EFL Learners’ Academic Buoyancy and Their Motivation and Interests

Studies, done under this subject, have been conducted since academic buoyancy does not solely focus on the academic arena; however, one’s personal wellbeing and academic buoyancy are delicately interwoven that they cannot be perceived as separable (Martin, 2013; Yun et al., 2018; Zhang, 2021). The reason lies in the fact when students are utterly motivated, they are more likely to be more tolerant of the setbacks, they may encounter in their process of learning, and they express a proactive way of coping with difficulties; hence, a solution to the problems can be raised. To be more precise, being buoyant has a positive impact on the way we behave in face of academic difficulties, for instance, when the received grades on the exams are poor, when stress levels increase owing to being under the pressure of daily routine, when they feel diffident because of their poor grades, for example, and suffer from lack of motivation and interests, and when students are given negative feedback on their poor performance. As a result, depending on the individuals’ abilities, the techniques they utilize for achieving their goals, and their emotional reactions to the goals, the level of their buoyancy might be different. Those who are motivated enough have been thought of as buoyant and they are less likely to abandon their goals if facing any hardships. So the level of motivation, accordingly, is ascribed to academic buoyancy. It is also stressed that when students are highly motivated, they feel buoyant during their studies through which their academic success increases and hardly ever do students’ acquisitiveness for learning curb.

## Takeaways

In the present review, the relationship between EFL students’ academic buoyancy and their motivation and interests has been discussed. These days, students are not viewed as passive members of the educational context, but active participants who help their teachers by giving feedback to make a friendly learning atmosphere. Without any doubt, in such an environment learners’ motivation, interest in learning, and academic achievements can be developed. Emphasis should be placed on the fact that these two variables, academic buoyancy and motivation, are highly correlated, meaning that an increase in one of which leads to a rise in another. There is nothing to compare with seeing a motivated student experience academic buoyancy as studying. Moreover, this study can be of great importance for authorities, striving hard to strengthen education infrastructures since they can provide teachers with seminars and workshops in which they are trained on how students’ motivation and academic buoyancy can be improved. Teachers can also find this study useful because their wellbeing highly depends on students’ satisfaction, which can be depicted in their academic buoyancy. Last but not least, it could be of great benefit for students themselves who sometimes suffer from lack of motivation to feel buoyant and they may be unaware of the fact that to what extent their emotions can be regarded as a contributory factor to their level of learning. Having said that, further studies are left for future avid researchers in some research areas. Firstly, what impacts both intrinsic and extrinsic motivation would have on students’ buoyancy is the topic that can delicately be discussed because the discrepancy between these two types of motivation has not yet been considered as far as the researcher’s knowledge goes. Secondly, most conducted studies have focused their attention on quantitative, retrospective, and self-reported data, even though some longitudinal studies based on introspective data and diaries should be carried out. Emphasis should additionally be put on the fact that most of the studies use random sampling which diminishes the generalizability; thus, non-random sampling can be utilized to scrutinize the impact of treatment on these constructs. Finally, from a cultural point of view, what has not been yet discussed in studies is the fact that both cultural and cross-cultural factors would be of utmost importance, regarding academic buoyancy. To sum up, the relationship between different aspects of teachers’ academic burnout and their academic motivation and interest should be probed with considerable attention in future studies in order to help teachers come up with new ideas for effective teaching.

## Author Contributions

All authors listed have made a substantial, direct, and intellectual contribution to the work and approved it for publication.

## Funding

This paper is supported by the Project of China National Committee for Translation & Interpreting Education (MTIJZW201925) and China Postdoctoral Science Foundation funded project (2019T120332).

## Conflict of Interest

The authors declare that the research was conducted in the absence of any commercial or financial relationships that could be construed as a potential conflict of interest.

## Publisher’s Note

All claims expressed in this article are solely those of the authors and do not necessarily represent those of their affiliated organizations, or those of the publisher, the editors and the reviewers. Any product that may be evaluated in this article, or claim that may be made by its manufacturer, is not guaranteed or endorsed by the publisher.
